# Pregelatinized Drum-Dried Wheat Starch of Different Swelling Behavior as Clean Label Oil Replacer in Oil-in-Water Emulsions

**DOI:** 10.3390/foods11142044

**Published:** 2022-07-11

**Authors:** Laura Roman, Mitchell R. Walker, Nicole Detlor, Janice Best, Mario M. Martinez

**Affiliations:** 1 Center for Innovative Food (CiFOOD), Department of Food Science, Aarhus University, AgroFood Park 48, 8200 Aarhus, Denmark; lroman@food.au.dk; 2 Department of Physics, University of Guelph, 50 Stone Road East, Guelph, ON N1G 2W1, Canada; 3 School of Engineering, University of Guelph, 50 Stone Road East, Guelph, ON N1G 2W1, Canada; mwalke15@uoguelph.ca; 4 Dawn Foods, 4370 Harvester Road, Burlington, ON L7L4X2, Canada; ndetlor@conestogac.on.ca (N.D.); janice.best@dawnfoods.com (J.B.)

**Keywords:** clean-label, rapid visco analyzer, mayonnaise, rheology, reduced-fat, stability, amylose

## Abstract

Drum-drying results in pregelatinized starch with relatively low starch fragmentation and a great ability to absorb water and swell at room temperature. However, the effect of the degree of cold particle swelling and the thickening potential of drum-dried starch on its suitability as oil replacer in low-fat oil-in-water emulsions has received little attention. In this work, the potential of three pregelatinized drum-dried starches with almost identical molecular structure (as measured by size exclusion chromatography) and Water Binding Capacity (WBC), but different swelling behavior, was investigated to replace up to 60% oil in a mayonnaise-like emulsion system. The microstructure, stability, and rheology of the oil-in-water emulsions were noticeably affected by the substitution of oil with a pregelatinized drum-dried starch paste. Specifically, reduced-fat emulsions presented smaller droplet-size, a higher consistency index and increased emulsion stability, especially against freeze-thaw cycles, compared to the control full-fat mayonnaise. Importantly, the differences in cold swelling behavior (rather than simply assessing WBC) greatly influenced the consistency index and stability of low-fat emulsions, and results showed that drum-dried starch particles with high swelling potential perform better as oil replacers.

## 1. Introduction

Oil-in-water emulsions in semi-solid form, such as mayonnaise and salad dressings, are widely used to enhance the flavor and mouthfeel of many dishes as toppings. Furthermore, they are also frequently used as a base for the development of other dressings [1]. However, they present oil contents typically higher than 65% (*w*/*w*) and therefore result in high-fat and high-calorie foods with the potential to increase the risk of health complications such as obesity [2], type-2 diabetes, cardiovascular diseases, and certain types of cancer [3,4]. According to the U.S. Census data and Simmons National Consumer Survey in 2020, almost 277 million Americans consumed mayonnaise or mayonnaise-type salad dressings, with a predicted growth of approximately 2.5% over the next four years [5]. Therefore, the development of oil-in-water emulsions in semi-solid form with reduced oil content could contribute to decrease the prevalence from suffering the non-communicable diseases attributed to a high fat diet.

Fat has a leading role in the texture and flavor of oil-in-water emulsions and its reduction can result in detrimental changes in the physicochemical, stability and organoleptic aspects of the final product. Furthermore, the thermodynamic instability of oil-in-water emulsions increases when their fat content is reduced below 60–65% [6]. Polysaccharide-based oil replacers have been shown to enhance the stability of oil-in-water emulsions by strengthening its rheology and decreasing the movements of oil droplets. In this regard, starches and other hydrocolloids such as β-glucan, xanthan, guar and locust bean gum were shown to have a unique ability to absorb water and develop viscosity in cold water, promoting the stabilization of the dispersed phase in mayonnaise-like emulsions [7,8,9,10]. Among them, starch represents the cheapest and one of the most abundant sources of hydrocolloid oil-replacers. However, starch is unable to swell and absorb water at cold temperatures in its native form due to its insoluble granular structure. Among the different techniques capable of modifying the supramolecular structure of native starch, hydrothermal processing is the most typical, such as drum-drying or extrusion [11,12], since they are long-recognized, clean-label and cost- and energy-efficient technologies that enable starch to absorb water and swell at room temperature. In this regard, extruded [13,14] and drum-dried starches/starchy flours [15] were shown to be effective oil replacers in oil-in-water emulsions by enhancing their stability.

Although drum-drying and extrusion both result in pregelatinized starch with the ability to absorb water and swell at room temperature, the end product might exhibit important differences depending on the shear, temperature and time histories, which is notably different between the two techniques. Firstly, extrusion results in a significantly higher molecular degradation due to the high shear attained in the screw-barrel assembly and the die [11], which could position drum-drying as a more interesting technology for this purpose. Secondly, preferential amylose leaching and, therefore, partial separation of amylopectin and amylose phases occurs during drum-drying [11,16], which could also affect the cold swelling, thickening and gelling behavior of drum-dried starches. However, the effect of the degree of cold particle swelling and thickening potential of drum-dried starch on its suitability as an oil replacer and the quality of low-fat oil-in-water emulsions has received little attention.

The objective of this study was to evaluate the effect of the thickening behavior of drum-dried wheat starches on the microstructure, rheology and stability of a mayonnaise-like oil-in-water emulsion model system, as well as the establishment of a rapid viscosity indicator of pregelatinized starch that enables the prediction of its suitability as an oil replacer in semi-solid oil-in-water emulsions. Furthermore, cold-swelling drum-dried starches were characterized in terms of molecular structure, swelling potential, water binding capacity, solubility and particle size to elucidate the underlying mechanisms responsible for emulsion rheology and physical stability.

## 2. Materials and Methods

### 2.1. Materials

Drum-dried pregelatinized starches from different wheat cultivars and batches were provided by Dawn Foods Inc (Toronto, ON, Canada). Pregelatinized starch samples were subjected to the same pregelatinization procedure through drum-drying, and three samples were selected according to a distinct cold viscosity behavior during RVA analysis. In this regard, samples were identified with the codes Pregel 1, Pregel 2 and Pregel 3, from lower to greater cold swelling behavior, as determined by the maximum cold peak viscosity in the RVA. Pregelatinized wheat starches were marketed to present 8% maximum packed-moisture and a pH of 5.0–6.5.

For the analysis of the starch’s fine molecular structure, dimethyl sulfoxide (DMSO, HPLC grade) and lithium bromide (ReagentPlus) were purchased from VWR (Radnor, PA, USA) and Beantown Chemical (Hudson, NH, USA), respectively. Sodium acetate (anhydrous, S210) and glacial acetic acid (A38S) were purchased from Fisher Scientific (Waltham, MA, USA). Sodium azide (≥99.5%, S2002) was purchased from Sigma-Aldrich (St Louis, MO, USA). Isoamylase (E-ISAMY, EC 3.2.1.68) from *Pseudomonas* sp. was purchased from Megazyme (Wicklow, Ireland).

For preparation of oil-in-water emulsions, whole liquid pasteurized egg (Burnbrae Farms, Brockville, ON, Canada), canola vegetable oil (Mazola, Mississauga, ON, Canada), ionized table salt, and pure white vinegar were obtained from a local supermarket. Potassium sorbate (AC29174) was purchased from Fisher Scientific (Waltham, MA, USA).

### 2.2. Methods

#### 2.2.1. Chain Length Distribution of Both Amylopectin and Amylose Molecules and Molecular Weight-Average Molecular Weight of Amylopectin

Amylopectin and amylose chain length distribution and amylose to amylopectin ratio (i.e., amylose content) were analyzed in duplicate after the debranching of starch molecules following the method detailed in Martinez et al. [17], which involves the solubilization of starch in DMSO before debranching with isoamylase enzyme. Analyses were performed using an Agilent 1260 size exclusion chromatography (SEC) system (Waldbronn, Germany) equipped with a refractive index detector (RI, 1260 RID, Agilent, Agilent Technologies, Waldbronn, Germany).

Data analysis and calculations to obtain SEC weight distribution plots were done as reported in Liu, Halley, and Gilbert [18], and Wang, Hasjim, Wu, Henry and Gilbert [19]. For debranched starches, the degree of polymerization (DP) of linear branches was calculated from the hydrodynamic volume (V_h_) using the Mark-Houwink equation [20] using a set of pullulan standards for V_h_ calibration. The length of short (A and B1) and long internal (B2, B3) amylopectin chains is denoted as X_Ap1_ and X_Ap2_, respectively, and the molar ratio of long to short amylopectin chains is presented as h_Ap2_/h_Ap1_. The amylose content was determined from the SEC molecular size distribution of debranched starch as the ratio of the area under the curve (AUC) of amylose chains relative to the AUC of both amylopectin and amylose chains.

**Figure 1:**
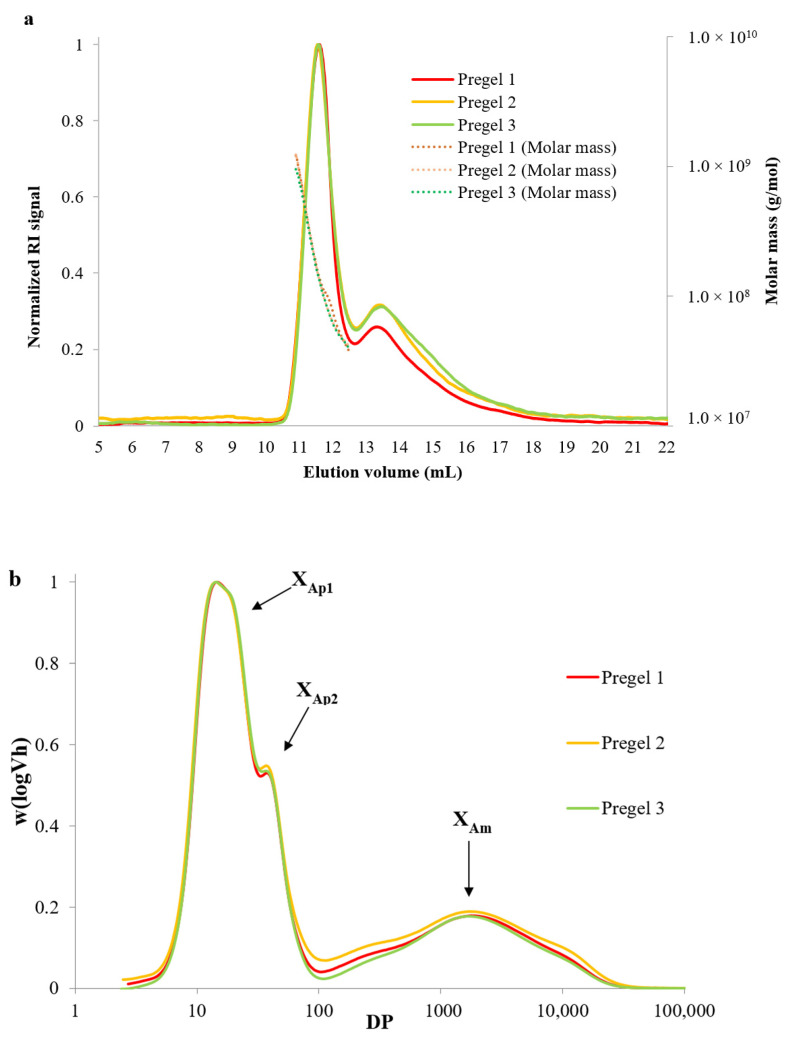
(**a**) Size exclusion chromatogram showing the normalized refractive index (RI) signal versus elution time for whole (branched) starch molecules, where two distinct peaks are observed corresponding to amylopectin (left) and amylose (right) molecules. The molar mass (g/mol) for amylopectin molecules is shown at the secondary axis. (**b**) Size exclusion chromatograms of debranched starch showing the unit chain length distribution of wheat starch samples. DP indicates degree of polymerization.

Furthermore, the weight-average molecular weight ($M_{w}$) of whole amylopectin molecules and its polydispersity (ratio between number-average molecular weight ($M_{n}$) and $M_{w}$) were measured using the aforementioned SEC-RI system coupled to a multi-angle light scattering detector (MALS, Dawn Heleos, Wyatt Technology, Santa Barbara, CA, USA) containing a K5-cell as previously described [21]. Briefly, starch was dissolved in a DMSO solution containing 0.5% (*w*/*w*) lithium bromide (DMSO/LiBr) at 80 °C overnight and centrifuged at 4000× *g* for 10 min. The supernatant was transferred into a SEC vial and then injected into GRAM 30 and 3000 columns (PSS GmbH, Mainz, Germany) connected in series. Data was analyzed with ASTRA software (version 4.72.03, Wyatt Technology Corporation, Goleta, CA, USA) fitting to a second-order Berry plot procedure. The specific refractive index increment (dn/dc) was assumed to be 0.066 mL/g and the second viral coefficient (A_2_) was assumed to be negligible. The obtained SEC chromatograms showing normalized RI signal versus elution volume are presented in Figure 1. Structural parameters of branched and debranched starch molecules are included as supplementary material (Table S1).

#### 2.2.2. Particle Size Distribution

The particle size and shape (sphericity) distributions of the wheat starches were analyzed in triplicate using dynamic image analysis (DIA) technology in a Dynamic Particle Analyzer Camsizer X2 equipped with an X-Dry module (Retsch Technology, Haan, Germany).

#### 2.2.3. Water Binding Capacity, Water Solubility Index and Oil Absorption Capacity

The Water Binding Capacity (WBC) and Water Solubility Index (WSI) of the starch samples was analyzed in triplicate using the AACC method 56.20-01 [22] with slight modifications and evaluated at 25, 50 and 90 °C. Briefly, 1.25 g of pregelatinized starchy material was weighed (weight of starch; W_s_) in a centrifuge tube (weight of tube; W_t_). Thereafter, 25 mL of milliQ water was added and vigorously vortexed to ensure sample hydration. The sample tubes were divided into three aliquots and soaked in three different water baths set at 25, 50 or 90 °C for 10 min. Afterwards, the heated tubes at 50 and 90 °C were allowed to cool down for 10 min in an ice water bath. After cooling, the tubes were centrifuged at 3000× *g* for 10 min. Supernatants in the tubes were carefully transferred into aluminum evaporating dishes of known weight (weight of evaporating dish; W_ed_) using disposable pipettes and the tubes containing the pellet residues (weight of tube + pellet; W_t+p_) were weighed to assess WBC. The evaporating dishes containing the supernatants were heated into an oven set at 105 °C for 24 h to evaluate the WSI. After overnight drying, the dishes containing the dry soluble residues (weight of evaporating dish + dry residue; W_ed+r_) were weighed and WSI was calculated. WBC and WSI values at 25, 50 and 90 °C were calculated using Equations (1) and (2), respectively.

(1) $$\mathrm{WBC}\;(g\;\mathrm{water}/g\;\mathrm{sample})=\frac{\left(\mathrm{Wt}+p\right)-\;\mathrm{Wt}\;-\;\mathrm{Ws}}{\mathrm{Ws}}$$

(2) $$\mathrm{WSI}\;(g\;\mathrm{solid}/100\;g\;\mathrm{sample})=\frac{\left(\left(\mathrm{Wed}+r\right)-\left(\mathrm{Wed}\right)\right)*100}{\mathrm{Ws}}$$

For the determination of oil absorption capacity (OAC), 2 mL centrifuge tubes were weighed (weight of tube; W_t_) and filled with 150 mg ± 0.02 of pregelatinized starches (weight of starch; W_s_) and 1.5 mL of vegetable oil. Samples were mixed with a thin rod to ensure that the pellet was fully dissolved. Tubes were then placed in a thermomixer and shaken at 500 rpm for 30 min. The tubes were then centrifuged at 3000× *g* and 4 °C for 10 min and the oil layer was removed using a pipette and the tubes were inverted onto a paper towel for a 25 min period to remove the excess oil. The tubes containing the residues were weighed (weight of tube + residue; W_t+r_). OAC (g oil/g sample) was determined in duplicate and calculated using Equation (3):

(3) $$\mathrm{OAC}\;(g\;\mathrm{oil}/g\;\mathrm{sample})=\frac{\left(W_{t+r}\right)-\left(W_{t}\right)-\left(W_{s}\right)}{W_{s}}$$

#### 2.2.4. Pasting Behavior

The pasting behavior of the starches was tested in duplicate in a Rapid Visco Analyzer (RVA 4800 Perten Instruments, Sydney, Australia). 4.5 g of each pregelatinized starch adjusted to 13% moisture was mixed into the RVA canisters with 25 mL of deionized water and mixed using a plexiglass stirring rod to ensure full hydration prior to RVA measurements. The pasting profile conditions were set as follows: rotation speed of 160 rpm and initial isothermal at 25 °C for 20 s, followed by a temperature increase at a constant rate of 1.4 °C/min for 50 min up to a temperature of 95 °C. Afterwards, the temperature was held constant for 10 min at 95 °C. The temperature was then decreased at 8 °C/min to 50 °C, which was then held for 2 min. From the viscosity profile of the starch pastes, the maximum and final apparent viscosities were calculated and reported as supplementary material (Table S2). Apparent viscosity was obtained at 160 rpm, which corresponded to a shear rate of 53 s^−1^.

#### 2.2.5. Preparation of Oil-in-Water Emulsions

Oil-in-water mayonnaise-like emulsions were prepared following the procedure of Roman, Martinez and Gomez [14], with slight modifications. 200 g of full-fat (Control) mayonnaise was prepared in 300 mL glass beakers using the following ingredients: 130 g vegetable oil, 62 g whole egg, 7 g vinegar, 0.9 g salt and 0.1 g of potassium sorbate. The ingredients were added in the following order: egg, salt and potassium sorbate, vinegar, and finally oil. For the reduced-fat mayonnaises, 30% and 60% of the vegetable oil was replaced by a starch-water paste freshly made on the day of the mayonnaise preparation with each of the three pregelatinized starches.

For the emulsions prepared with 30 and 60% reduced oil concentrations, starch-water pastes were used to replace the vegetable oil. These pastes were made at a 1:5 starch-water ratio based on the optimum WBC of the starches and the absence of syneresis according to previous testing. Briefly, 5 g ± 0.01 of the pregelatinized starch was mixed with 25 mL (1:5 ratio) of deionized milliQ water and then stirred with a glass rod until no lumps were visible, ensuring the adequate mixing and hydration of the starch material.

The oil-in-water emulsion was formed using an IKA T-18 Ultra Turrax digital homogenizer operating at 14,000 rpm for 2 min. After homogenization, the control and reduced-fat mayonnaises were transferred to sealed glass containers and stored at 4 °C until further testing. The composition of the emulsion is summarized in Table 1. The full fat mayonnaise was labeled as control, while reduced-fat mayonnaises were labeled according to the starch type (Pregel 1, Pregel 2 and Pregel 3) used for oil replacement followed by the oil replacement level (30% or 60%). The seven different mayonnaises were prepared in duplicate.

#### 2.2.6. Microstructure of the Full Fat and Reduced-Fat Mayonnaises

Twenty-four hours after the emulsions were prepared, the microstructure was observed with a Dm750 microscope (Leica Microsystems, Wetzlar, Germany) with a 40× objective magnification. A small droplet of each mayonnaise sample was placed on a glass microscope slide and covered with a glass strip. A one kg weight was then used to compress the covered microscopes slides for 10 min to ensure a uniform thickness. Two slides of mayonnaise samples were tested for each formulation, and micrographs were taken at least twice in two random points of each microscope slide. Images of emulsions were analyzed using ImageJ software (National Institute of Health, Bethesda, MD, USA) after image binarization to obtain particle analyses on the number of oil droplets, average area of the oil droplets, and total area within the emulsion comprised by oil droplets. The studied area was 18.72 × 89.04 μm^2^ for each image captured.

#### 2.2.7. Steady-State Flow Behavior of Oil-in-Water Emulsions

Rheological properties of the oil-in-water emulsions were evaluated 24 h after mayonnaise making in a Discovery Hybrid Rheometer 3 (DHR-3) (TA Instruments, New Castle, USA) equipped with a Peltier plate for temperature control at 25 °C. Rheological tests were carried out using a 20 mm diameter parallel plate geometry with 100 grit medium sandpaper on the surface of the plates to avoid slippage effects. Samples were placed on the bottom plate geometry, the gap was adjusted to 1 mm and the exposed edges were covered with vaseline oil. After loading, the samples were let to rest for 6 min before measurement.

To assess steady-state flow behavior of the oil-in-water emulsion and obtain the shear stress versus shear rate data, the shear rate was logarithmically increased from 1 to 100 s^−1^ (up curve), then the shear was maintained at 100 s^−1^ for 60 s, followed by a shear rate reduction from 100 to 1 s^−1^ (down curve). Data was recorded for each point when the steady state was reached. Flow behavior evaluated between these shear rate values is assumed to be in the range for chewing and swallowing [23]. Finally, the data of the upper curve were adjusted to the Ostwald de Waele equation (Equation (4)) [9,10,14]:

σ = Κ × (ϒ)^n^ (4)

where σ is the shear stress (Pa), ϒ is the shear rate (s^−1^), K is the consistency index (Pa·s^n^) and n is the flow behavior index (dimensionless).

The relative thixotropic area was calculated as the area difference between the up and down curves divided by the area under the upper curve. This calculation for the hysteresis loop allows for the accurate comparison of breakdown within structures in systems with differing viscosities [24]. The rheological properties of each formulation were evaluated at least in duplicate.

#### 2.2.8. Emulsion Short-Term Stability, Long-Term Stability and Freeze-Thaw Stability

Emulsion stability tested under different storage conditions was performed in duplicate following a modified method of Mun et al. [8]. Emulsions aliquots of 15 g ± 0.01 g (W_o_) of each of the formulations were placed into 50 mL centrifuge tubes and stored under three different conditions to promote destabilization. For short-term emulsion stability, the tubes were stored at 4 °C for 24 h, placed at room temperature for 1 h, and then heated at 80 °C for 30 min in an air oven to promote the destabilization of the emulsion. Samples were centrifuged at 4000× *g* for 10 min, after which the separated layer was removed and weighed (W_1_). Emulsion stability (%) was calculated using Equation (5):

(5) $$\mathrm{Emulsion}\;\mathrm{Stability}=\frac{W_{o}-{\;W}_{1}}{W_{o}}\times 100$$

Similarly, 15 g of emulsion samples were also kept at 4 °C for 31 days (one month), after which the long-term stability was also tested as mentioned above for short-term stability. Furthermore, freeze-thaw stability was assessed after storing mayonnaise aliquots at −20 °C for 24 h, then thawed for 1 h, and centrifuged as indicated above to collect and weigh the amount of the separated oil layer. The freeze-thaw emulsion stability was calculated using the emulsion stability equation as mentioned above (5).

#### 2.2.9. Statistical Analysis

Differences among physicochemical properties of starches and emulsions were studied by a one-way analysis of variance (one-way ANOVA) using the Fisher’s least significant difference (LSD) to describe means with 95% confidence intervals. Statistical analysis was performed with Statgraphics Centurion XVI software (Statpoint Technologies, Inc., Warrenton, VA, USA).

## 3. Results and Discussion

### 3.1. Molecular Features of Drum-Dried Wheat Starches

The molecular size distributions of branched and debranched starch polymers used as part of the reduced-fat emulsions were characterized using size exclusion chromatography and normalized elution profiles were reported in Figure 1. The normalized elution profile (refractive index signal) of whole starch molecules as well as the molar mass distribution is included in Figure 1a. The weight-average molecular weight (M_w_) and polydispersity of amylopectin from the starches is shown in Table S1. Amylopectin from the different molecules did not show significant differences in M_w_ (~2.1 × 10^8^ Da) and polydispersity (ranging from 1.9 to 2.1). Slightly higher values for M_w_ of amylopectin from regular wheat containing 27% amylose were reported (3.1 × 10^8^ Da) [25], which could be attributed to the different cultivars or due to the slight molecular fragmentation occurring during drum-drying [11,26,27]. Specifically, drum-drying results in a significantly lower decrease of starch M_w_ compared to extrusion, which typically results in pregelatinized starches with a relatively low solubility index, as will be discussed in Section 3.2. For example, Hayes, Okoniewska, Martinez, Zhao, and Hamaker [26] reported a negligible reduction in M_w_ of native wheat starch from 1.97 × 10^8^ to 1.94 × 10^8^ Da after drum-drying, whereas the M_w_ decreased to 0.86 ×10^8^ after extrusion.

Typical chain length distributions of debranched starch molecules (Figure 1b) show bimodal peaks representing short (peak DP~14) and long (peak DP~36) amylopectin chains as well as amylose chains (DP~100–10,000) [19]. Results for the amylose and amylopectin fine structural parameters extracted from the chromatograms are reported in Table S1, and minimal differences were observed between the three wheat starches studied. Wheat starches presented similar amylose content with values ranging between 27.6% and 28.7% total content. These results were consistent with the findings of other studies on the amylose to amylopectin ratio of wheat starches, suggesting that the average amylose content ranged between 20 and 30% total weight content [28]. Furthermore, the average chain length of amylose molecules (X_Am_) ranged from 1625 to 1738 DP, being slightly but significantly longer for Pregel 1 wheat starch compared to Pregel 2 and 3. The amylose content and its average length also agrees with those reported by Martinez et al. [17] for wheat starch containing 25.6% amylose with an average chain length of 1713 DP. In addition, no significant differences existed between the ratios of long to short amylopectin chains (h_Ap2_/h_Ap1_) and amylose to short amylopectin chains (h_Am_/h_Ap1_), with all samples clustered around values in a range of 0.54–0.57 and 0.17–0.18, respectively. This would indicate that all samples had a similar proportion of long to short chains and amylose to amylopectin chains. Negligible differences were observed in the amylopectin peak ratios between the starch samples and the degrees of polymerization at maximum amylopectin peaks for short (A + B1) chains, although Pregel 1 showed significantly longer (B2 + B3) chains, as represented by X_Ap2_ values. These values are similar to those found by Martinez et al. [17] with 13.0 and 39.0 for another variety of wheat starch. It is worth noting that the slightly longer long population of amylopectin chains and amylose chains of Pregel 1 starch should not be expected to be more prone to retrograde due to their minimal difference in chain length.

### 3.2. Particle Size and Sphericity Distribution, Solubility and Liquid Holding Capacity of Drum-Dried Wheat Starches

**Figure 2:**
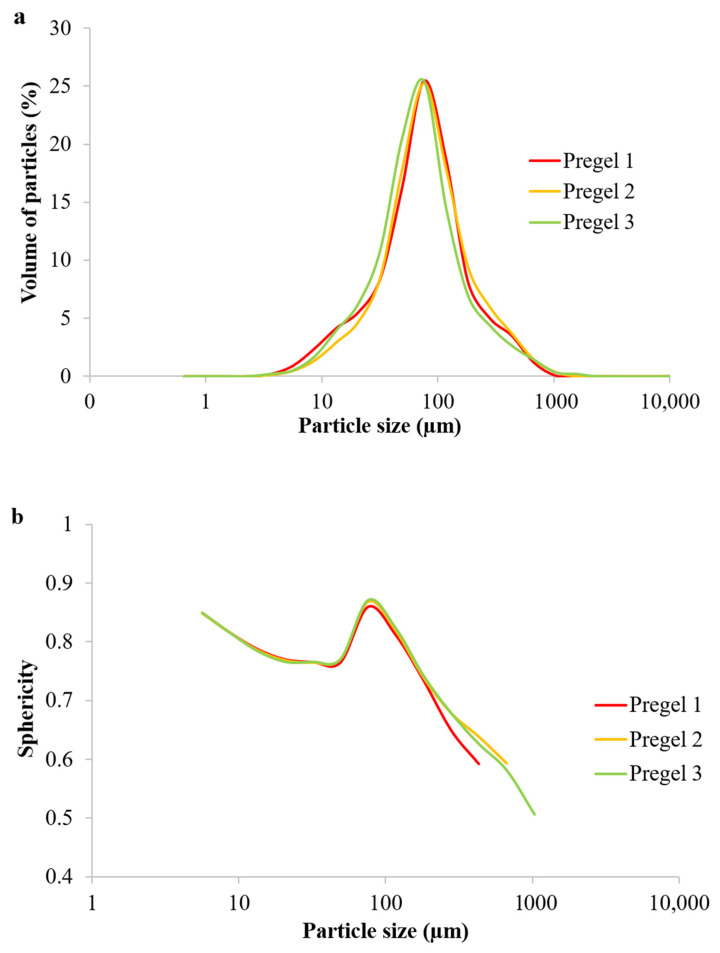
Particle size distribution (**a**) and sphericity (**b**) of particles in the pregelatinized wheat starches.

Little differences in the size distribution and sphericity of the particles (Figure 2) after drum drying were observed. All of the samples showed a maximum sphericity of 0.87, which occurred in starch particles with a size of ~76.1 μm (where the maximum volume of particles was also found). It is worthy of note that a perfect sphere will present a sphericity of 1. It is noteworthy that Pregel 1 starch presented humps on both sides of the Gaussian-like particle size distribution (Figure 2a), indicative of slightly more agglomeration than in the other wheat counterparts. Pregel 1 starch also presented particles of 170 µm and above (which still represents a large population of particles by volume according to Figure 2a) with significantly lower sphericity (Figure 2b).

**Figure 3:**
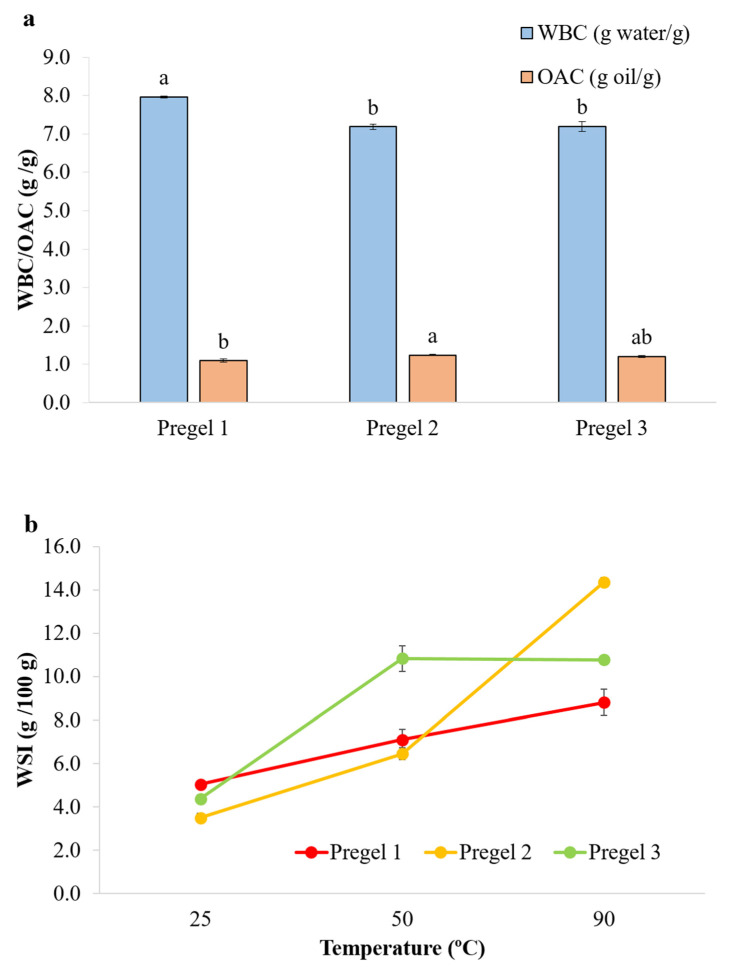
(**a**) Water binding capacity (WBC, g/g) and oil absorption capacity (OAC, g/g) of wheat starches at room temperature. Different lowercase letters above each bar denote significant differences. (**b**) Water solubility index (WSI, g/100 g) of wheat starches at three different temperatures.

WSI, WBC and OAC of drum-dried wheat starches are summarized in Figure 3. At 25 °C, minimal differences were observed in OAC and WSI among the different starch samples, whereas Pregel 1 showed slightly but significantly higher WBC (Figure 3a). WSI behavior was also analyzed at 50 and 90 °C (Figure 3b). All the starches denoted a significant increase in WSI when heated from 25 to 50 and 90 °C. This suggests that the amorphous and dense gelatinized starch particles were more accessible to hydration with increasing temperature, especially at 90 °C, where there was a fast solubilization of the particles. A faster diffusion of the water to the inner part of these dense amorphous particles is expected at higher temperatures, with the softening of the starch leading to a faster access to the inner part of the particles. This would result in a higher WBC and WSI of the particles. It is noteworthy that Pregel 1 presented lower propensity for solubilization with increased temperature, which could suggest greater molecular entanglement restricting the solubilization and swelling of starch particles [29]. This mechanism is supported by the presence of visibly more amylopectin double helices on Pregel 1 than Pregel 2 and Pregel 3, as shown by the endotherm peak (~54.7 °C) in the DSC thermograms (Figure S1). At 90 °C, Pregel 2 presented the highest WBC (data not shown) and WSI, although results at this temperature are not expected to affect the emulsion behavior, as the oil-in-water emulsions were not heated during processing. The WSI values found in this study for the drum-dried starches varied from 3.71–5.04% at 25 °C and 5.4–10.8% at 50 °C, while those reported by Colonna and coworkers [11] were significantly higher, ranging from 13.6–18.9% and 24.6–36.9% for 25 and 50 °C, respectively. The differences in the values found for the WSI can be related to the different conditions selected during drum-drying, suggesting that a harsher drum-drying treatment was performed in their study. Similarly, Fritze [30] reported solubility values of 10.5–13.4% in cold water in drum-dried maize starch.

### 3.3. Pasting Profile of Drum-Dried Wheat Starches

**Figure 4:**
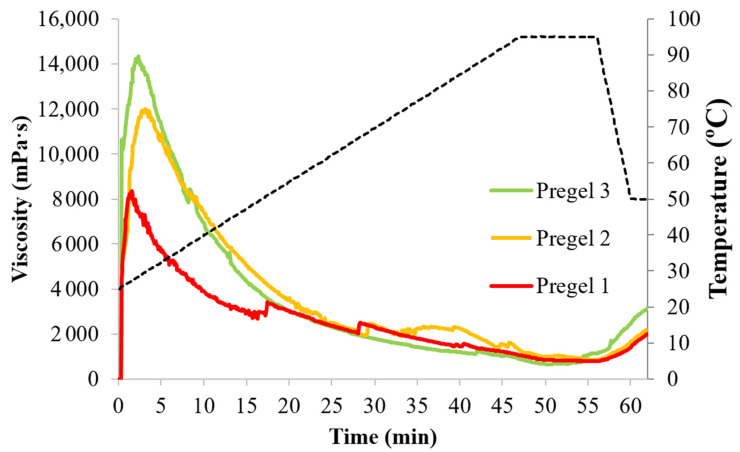
Pasting profiles of the pregelatinized wheat starches during a temperature profile up to 95 °C. Black broken lines represent the temperature profile.

The pasting profile of the drum-dried starches is shown in Figure 4. The pasting profiles agree with the ones previously observed for extruded or drum-dried starches [26,29,31]. All starches developed an instant peak viscosity at 30 °C (cold peak viscosity) and lacked the typical viscosity peak attributed to starch gelatinization, suggesting a complete starch gelatinization during drum-drying. Pregel 1, however, still presented remnants of amylopectin double helices in the DSC thermographs (Figure S1), suggesting either/both non-complete starch gelatinization and/or amylopectin retrogradation occurring during drum-drying. Drum-drying is relatively slow, therefore starch molecules have the possibility of re-associating [32]. In any case, all samples were able to absorb water and thicken without the need of heating.

The cold peak viscosity (Figure 4 and Table S2), indicator of the starch thickening ability in cold, followed the order of Pregel 3 > Pregel 2 > Pregel 1, with maximum viscosities of 14,340, 12,833, and 8360 mPa·s, respectively. Despite the similar degree of starch fragmentation during drum-drying (Table S1), Pregel 1 exhibited a significantly lower cold viscosity, which could be attributed to a greater molecular entanglement of the particles restricting particle swelling and amylose leaching. In fact, Pregel 1 presented remnants of amylopectin double-helices detected by DSC (Figure S1) and the slowest WSI increase upon heating (Figure 3). This event would also explain the lower breakdown (difference between maximum peak viscosity and minimum viscosity upon heating) of Pregel 1, compared to Pregel 2 and Pregel 3. Remarkably, Pregel 3 exhibited a significantly greater cold peak viscosity than Pregel 2. This phenomenon may be interpreted on the basis of starch-water interactions. During drum-drying, where no shear is applied to the swollen granules, amylose and amylopectin components might separate to a different extent due to preferential amylose leaching. Leached amylose, therefore, separates from the amylopectin-rich fraction and might crystallize in its volume fraction [16]. Remarkably, amylose leaching critically determines granular/particle swelling [33], which could explain the distinct pasting profiles between Pregel 2 and Pregel 3 samples. Presumably, Pregel 3 underwent less amylose leaching during drum-drying, which enhanced the maximum swelling of drum-dried particles during the pasting cycle in the RVA. This phenomenon is also supported by the greatest WSI increase of Pregel 3 upon heating from 25 °C to 50 °C, which would only account for readily soluble amylose that was not previously retrograded. Nevertheless, further studies are needed to confirm this phenomenon.

The final viscosity of the starches was significantly lower than the viscosity attained during cold swelling. Among samples, Pregel (3115 mPa·s) exhibited a significantly higher final viscosity than Pregel 1 (1969 mPa·s) and Pregel 2 (2185 mPa·s) samples. Doublier, Colonna, and Mercier [29] correlated the reduction in final viscosity and setback with the fragmentation of some amylose chains due to shear occurring during processing, which might make these molecules lose the ability to associate and retrograde effectively during cooling. However, our reported starch did not differ in molecular fragmentation. These differences might also be explained by a lower amylose leaching of Pregel 3 during drum-drying compared to Pregel 1 and Pregel 2 samples, as explained before, since only amylose that did not retrograde previously during drum-drying would contribute to the short-term viscosity rise in the RVA upon cooling.

### 3.4. Microstructure and Rheological Properties of the Oil-in-Water Emulsions

**Figure 5:**
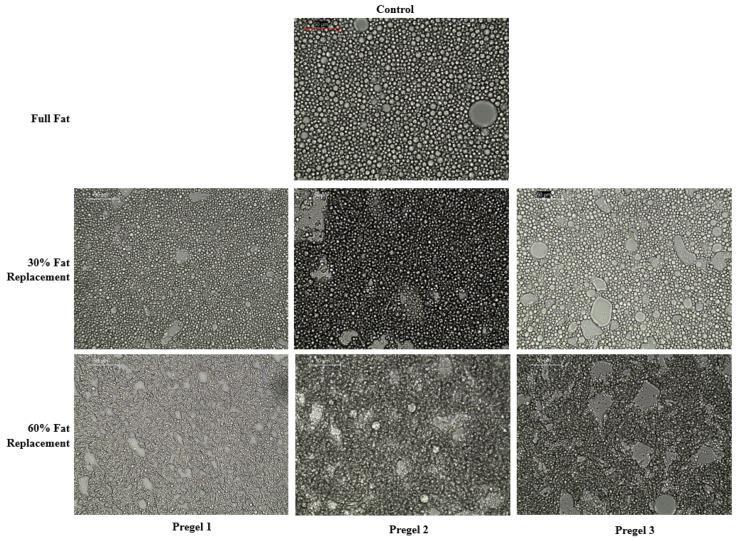
Optical microscopy images of mayonnaise like emulsions taken at 40× magnification 24 h after their making. The scale bar indicates a size of 50 μm.

The oil droplet size distribution, number of oil droplets and flow rheological parameters of oil-in-water emulsions are summarized in Table 2. Furthermore, the microstructure of the emulsions is also shown in Figure 5.

Full-fat (control) emulsion showed a closely packed distribution of oil droplets, occupying most of the emulsion volume evenly. A reduction in the oil content, which in this case also coincided with the incorporation of drum-dried starch, led to the lower average size of the oil droplets. This effect was significantly visible for all of the drum-dried starch types, and no significant differences were observed between the starch types for each replacement level. There was also a noticeable decrease in the total area covered by oil droplets at increasing the levels of oil substitution (Table 2), displaying more empty areas that were most probably occupied by starch particles (Figure 5). It is well known that the size reduction of droplets during homogenization depends on the balance between two opposite phenomena: droplet disruption and droplet coalescence. This balance is strongly driven by the viscosity and interfacial tension of the aqueous phase [34]. In this study and in terms of emulsion formation and stabilization, pregelatinized starch was added with the aim of stabilizing the emulsions based only on its thickening ability, since egg yolk (i.e., emulsifier) is presumed to be sufficient to significantly decrease the interfacial tension at the interface of the oil droplets. Thus, microstructure differences of the produced oil-in-water emulsions should be related to the volume fraction and viscosity of the continuous phase. Drum-dried starch, which possesses high water binding capacity, forms a strong and viscous continuous network that might inhibit the oil droplets from coalescing into larger droplets. Furthermore, the positive effect that a more viscous continuous phase could have on the homogenization and stabilization rate favoring smaller droplets should not be ruled out, for example, by decreasing the minimum shear rate needed to decrease the droplet size in the reduced-fat mayonnaise. It is noteworthy that mayonnaise possesses non-Newtonian flow (as shown in Table 2 and discussed below), with a high oil volume fraction as a dispersed phase, which is expected to result in significant re-coalescence during emulsification [34]. As the volume fraction of the disperse phase decreases, it is less plausible that re-coalescence takes place during emulsification. This occurrence, together with the higher viscosity of the continuous phase, could explain, in all likelihood, the smaller droplets in reduced fat-emulsions as compared to the full fat control. The microstructures for the reduced-fat emulsions were consistent with previous findings in which the droplet size was reduced with oil replacement by chemically modified starch or pregelatinized extruded flours [8,14,35].

The Ostwald de Waele or Power-law model was used to model the flow behavior of the emulsions (R-squared values ≥ 95%). The results are presented as consistency index, K, and the flow exponent index, n, which reflects the deviation of the fluid flow from the Newtonian (Table 2).

All of the emulsions behaved as non-Newtonian fluids, with a flow behavior index value firmly in the shear-thinning region (*n* << 1), with n values between 0.20 and 0.40 [36]. The n value significantly decreased for 60% fat-replacement emulsions (Pregel 2 and 3), indicating stronger shear-thinning behavior, whereas no differences were found between the 30% fat-replacement and the full-fat emulsion. The decrease in flow behavior index with increasing level of oil replacement in the emulsions was also observed by Roman et al. [14], with n values ranging from 0.20 to 0.24. Furthermore, Aganovic, Bindrich, and Heinz [37] also found that the flow exponent decreased for high-pressure homogenized oil-in-water emulsions, which correlated to the smaller-droplet size in those emulsions.

In contrast, the flow consistency index, K, significantly increased for the reduced-fat emulsions, especially at the 60% replacement level, likely due to the immobilization of the droplets by the continuous phase containing drum-dried starch particles. Increases in K value in the emulsion indicates a more pronounced viscous response to the applied shear forces and thus a stronger structure [38]. These results agreed with those reported by Mun et al. [8] and Roman et al. [14], who replaced the oil with a paste made of other modified starches. For 60% replacement, the increase in K was significantly higher for Pregel samples 2 and 3, which denoted the highest consistency, whereas the Pregel 1 sample denoted the lowest consistency. The lower consistency of the Pregel 1 (60%) emulsion could be ascribed to the low ability of Pregel 1 particles to hydrate, swell, and develop viscosity, because of higher molecular entanglement, as discussed in Section 3.3. The slightly (non-significant) higher consistency of Pregel 3 emulsions compared to their Pregel 2 counterpart could also be explained based on the higher capacity of Pregel 3 particles to swell.

**Figure 6:**
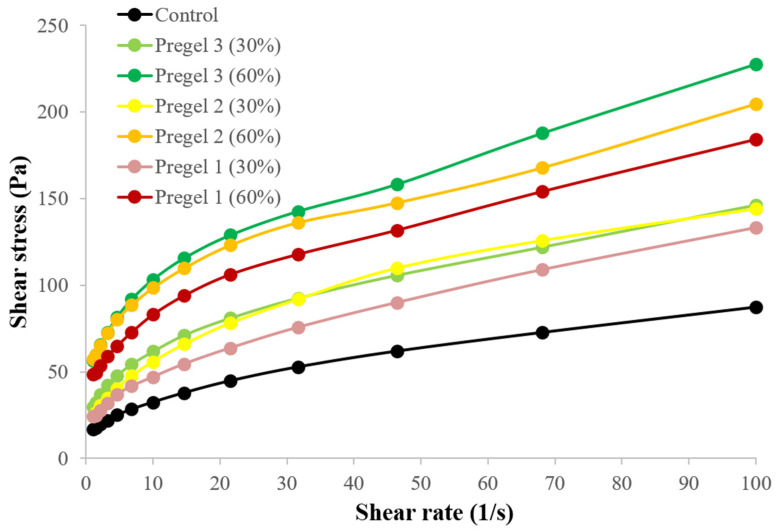
Flow behavior of control and reduced-fat (30 and 60%) oil-in-water emulsions under steady-state conditions.

Although the rheological behavior of mayonnaises is often adjusted to the Power-law equation due to its higher R-squared values [9,10,14,37], the presence of a yield stress (τ_0_), was observed in all the emulsions (Figure 6). The yield stress was significantly higher for the reduced-fat samples than for the full-fat emulsion [8,23], especially for Pregel 2 and Pregel 3 at 60% replacement. The presence of a yield stress determines the stability of emulsions in low stress conditions, such as those happening during storage and transportation, the spreadability or ability to adhere to the surfaces at the time of utilization and the thickness or mouthfeel during mastication, among others [8,23,34].

Shear-thinning behavior is a determinant of important characteristics in the mayonnaise such as mouth feel and flowability [38,39]. Hence, the increased consistency, yield stress and slight decrease in the shear-thinning behavior with the incorporation of the pregelatinized starch suggests that the starches were highly effective for replacing oil in oil-in-water emulsions without compromising the quality of the final product.

The presence of a hysteresis loop was used to determine the degree of time dependency in the flow behavior of the emulsion and was quantified as relative thixotropic area (Table 2). For the hysteresis loop test, the share rate was increased from zero to a maximum value (Figure 6), and then decreased back to zero in the same fashion. This test suggests how rapidly the sheared structure can adapt and rebuild its original structure after removal of the shear forces [40]. All of the emulsions exhibited time-dependence and behaved as thixotropic fluids, with their apparent viscosity (or the corresponding shear stress) decreasing with the time of shearing. The full-fat and 30% reduced-fat emulsions showed the lowest thixotropic area, which may be related to their more closely-packed droplet distribution. For 60% fat-replacement, the emulsion made with Pregel 1 starch presented the lowest thixotropic behavior compared to the rest of starches, with Pregel 2 starch resulting in the largest thixotropic area. Thus, a higher oil replacement level tended to increase the thixotropic behavior [14]. In general, it was found that the reduced-fat emulsions with the higher consistency index also resulted in higher thixotropy. These results may indicate that the notable thickening of the continuous phase by the starch particles could inhibit/reduce the movement of oil droplets in the dispersed phase, increasing the time required to re-stabilize the original structure after the removal of the shear force. In this regard, in high fat emulsions, oil droplets are packed together, and their movement is strongly impeded, while for reduced-fat emulsions, the movement of the droplets is usually reduced by including polysaccharides with strong thickening ability and water binding potential [41].

### 3.5. Stability of the Oil-in-Water Emulsions

The stability of the oil-in-water emulsions under different storage conditions are reported in Table 3. Emulsion instability is usually associated with droplet coalescence, flocculation and creaming phenomena. After one-day storage and subsequent heating at high temperature (denoted as short-term stability), all of the reduced-fat emulsions were 100% stable and, interestingly, low-fat emulations showed more enhanced stability than the full-fat control, the latter displaying an instability of 93.8%. Similarly, after one-month of storage (long-term stability), the full-fat emulsion was the least stable (77.2% stability), while all the reduced-fat emulsions showed stability higher than 94%. Among the low-fat emulsions, Pregel 1 starch resulted in small but significantly lower long-term stability for both levels of replacement which, by all likelihood, is associated with its lower thickener potential, as shown by its lower consistency index, yield stress and cold-water viscosity. In any case, the increased stability cannot be fully related to the percentage of oil in the emulsion, as minimal differences were found between 30% and 60% substitutions, with only a ~5% increase in emulsion stability (from 94.4 to 99.6% stability, respectively). This occurrence indicates that the presence of pregelatinized starch was very effective in maintaining the structure of the emulsion, even after long storage times. In the reduced-fat mayonnaises, the increased rigidity of the structure, due to the fat replacer in the continuous phase, together with the small droplet size could have created strong gel-like interactions, impeding droplet movement and increasing the stability of the system. These results prove that all of the starches were very effective thickeners, creating a more stable oil-in-water emulsion. However, attention to the cold hydration and swelling ability of drum-dried starches should be paid, since it can critically influence the rheology properties of low-fat emulsions and stability. These results agreed with other studies on emulsion stability which concluded that emulsion stability increased with oil replacement levels [9,13,42].

Most oil-in-water emulsions are susceptible to increased destabilization after freezing and subsequent thawing, mainly due to the large expansion of ice crystals within the structure as the water freezes. This expansion not only damages the structure of the emulsion, but also forces the oil droplets closer together, which increases the risk of coalescence and emulsion destabilization [43,44]. In agreement with previous findings [14,35], this research found that the control full-fat mayonnaise was the least stable during the freeze-thaw process, with freeze-thaw stability increasing as the level of oil substitution increased. Most noticeable was the increase in stability from the full-fat mayonnaise to 60% reduced-fat emulsions, with stability increasing from 51.5% (full-fat) to values between 91.9–93.2% for all 60% replacement. Although few differences in stability were observed between starches, Pregel 3 (60%) denoted the highest freeze-thaw and long-term stability, which could be associated with its higher consistency index and swelling potential. As a whole, these combined characteristics probably resulted in the higher viscosity of the continuous phase, reducing the mobility and destabilization of the small, dispersed oil droplets due to possible flocculation and coalescence phenomena [34].

## 4. Conclusions

It can be concluded that the starches modified through drum-drying could serve as effective oil replacers for oil-in-water emulsion-like products. The microstructure, stability, and rheology of the oil-in-water emulsions were noticeably affected by the substitution of oil with pregelatinized drum-dried starch paste. All reduced-fat mayonnaises exhibited enhanced emulsion stability compared to the control full-fat emulsion, especially against freeze-thawing processing. The average size of the dispersed oil droplets decreased with the use of drum-dried starches, likely due to the increase of the viscosity of the continuous phase. Importantly, although the three drum-dried starches showed almost identical molecular features and WBC, they exhibited different swelling and solubilization behaviors during pasting, which was attributed to potential differences in the presence of molecular entanglement (e.g., amylopectin double helices) and leached amylose after drum-drying. These swelling differences found in the RVA cycle seemed critical to achieving a successful replacement of oil in terms of emulsion stability and rheological behavior. This would indicate that pasting behavior is important to consider for the selection of starches as oil replacers (rather than simply assessing WBC), as too low a viscosity in cold water could represent a limiting problem. Although out of the scope of this work, further sensory trials could complement these findings by adding more descriptors that are not provided by analytical instruments, such as mouthfeel of consumer acceptability.

## Figures and Tables

**Table 1:** Composition of oil-in-water emulsions based on the weight of the ingredients used in grams. Composition are based on 200 g of total emulsion formulation.

Ingredient	Full Fat (Control)	30% Reduced Fat	60% Reduced Fat
Vegetable Oil	130.0	91.0	52.0
Whole liquid Egg	62.0	62.0	62.0
White Vinegar	7.0	7.0	7.0
Salt	0.9	0.9	0.9
Potassium Sorbate	0.1	0.1	0.1
Starch/water paste *	0.0	39.0	78.0

* 1:5 pregelatinized wheat starch:water ratio. Pregel 1, 2 and 3 were used to prepare the reduced-fat.

**Table 2:** Average area, total area of droplets, number of droplets and rheological parameters for full-fat and reduced-fat oil-in-water emulsions.

Sample	Number of Droplets	Total Area of Droplets (μm^2^)	Average Area (μm^2^)	K Index (Pa·s^n^)	n	Thixotropic Area (%)
Control	3976 ± 121 [a]	27,533 ± 3042 [a]	7.2 ± 0.5 [a]	15.7 ± 1.2 [d]	0.36 ± 0.02 [ab]	4.3 ± 0.3 [d]
Pregel 1 (30%)	5438 ± 621 [a]	21,255 ± 2461 [abc]	3.6 ± 0.1 [bc]	22.8 ± 1.4 [c]	0.37 ± 0.00 [ab]	4.1 ± 0.6 [d]
Pregel 2 (30%)	4817 ± 663 [a]	14,876 ± 1356 [de]	3.1 ± 0.4 [bc]	22.6 ± 2.3 [c]	0.40 ± 0.01 [a]	3.3 ± 0.7 [d]
Pregel 3 (30%)	5323 ± 896 [a]	24,520 ± 7128 [cd]	4.6 ± 0.6 [b]	26.5 ± 1.2 [c]	0.36 ± 0.01 [ab]	5.7 ± 0.1 [c]
Pregel 1 (60%)	4578 ± 361 [a]	10,894 ± 9642 [d]	2.2 ± 0.6 [c]	39.2 ± 0.3 [b]	0.32 ± 0.01 [bc]	6.1 ± 0.6 [c]
Pregel 2 (60%)	6166 ± 386 [a]	15,681 ± 2552 [de]	2.5 ± 0.3 [c]	52.5 ± 1.3 [a]	0.27 ± 0.01 [c]	13.7 ± 0.1 [a]
Pregel 3 (60%)	6012 ± 462 [a]	19,299 ± 1688 [bc]	3.2 ± 0.4 [bc]	53.0 ± 2.4 [a]	0.26 ± 0.0 [c]	9.2 ± 0.1 [b]

Values ± standard deviations with different letters in the same column are significantly different with *p* < 0.05. K index, consistency index; n, flow behavior.

**Table 3:** Stability (%) of the oil-in-water emulsions after varying storage time conditions and freeze-thaw processing.

Sample	Short-Term Stability (%)	Long-Term Stability (%)	Freeze-Thaw Stability (%)
Control	93.8 ± 0.1 [b]	77.2 ± 0.3 [f]	51.5 ± 0.1 [g]
Pregel 1 (30%)	100.0 ± 0.0 [a]	94.4 ± 0.1 [e]	75.8 ± 0.4 [d]
Pregel 2 (30%)	100.0 ± 0.0 [a]	96.2 ± 0.0 [c]	75.4 ± 0.1 [e]
Pregel 3 (30%)	100.0 ± 0.0 [a]	94.8 ± 0.0 [d]	73.3 ± 0.1 [f]
Pregel 1 (60%)	100.0 ± 0.0 [a]	99.3 ± 0.0 [b]	92.1 ± 0.0 [b]
Pregel 2 (60%)	100.0 ± 0.0 [a]	99.5 ± 0.0 [ab]	91.9 ± 0.1 [c]
Pregel 3 (60%)	100.0 ± 0.0 [a]	99.6 ± 0.1 [a]	93.2 ± 0.1 [a]

Values ± standard deviations with different letters in the same column are significantly different with *p* < 0.05.

## Data Availability

Data is contained within the article or Supplementary Material.

## Supplementary Materials

The following supporting information can be downloaded at: https://www.mdpi.com/article/10.3390/foods11142044/s1, Table S1: Molecular weight, polydispersity index, amylose content, amylose and amylopectin chain length and ratios between amylose and amylopectin chains of the pregelatinized drum-dried starches; Table S2: Peak maximum viscosity and final viscosity obtained from the pasting profiles of the pregelatinized drum-dried starches; Figure S1: Differential scanning calorimetry curves of drum-dried starches scanned at 10 °C/min from 25 to 95 °C after 24 h of hydration with a 1:3 starch:water ratio. Arrow indicates the presence of an endothermal transition in Pregel 1 starch presumably corresponding to the gelatinization of amylopectin double helices as previously reported for native wheat starch [45].
