# Supplementary material for: Pregelatinized Drum-Dried Wheat Starch of Different Swelling Behavior as Clean Label Oil Replacer in Oil-in-Water Emulsions
Source: Foods. 2022 Jul 11;11(14):2044. doi: 10.3390/foods11142044 (PMC9318365; doi:10.3390/foods11142044)
Supplement: Supplementary file 1 [file foods-11-02044-s001.zip › foods-1782695-supplementary.pdf]

Pregelatinized drum-dried wheat starch of different swelling behavior as clean-labeled oil replacers in oil-in-water emulsions

Laura Roman, Mitchell R. Walker, Nicole Detlor, Janice Best, Mario M. Martinez

Table S1. Molecular weight, polydispersity index, amylose content, amylose and amylopectin chain length and ratios between amylose and amylopectin chains of the pregelatinized drum-dried starches.

Sample	M_w (× 10^8 g/mol)	Polydispersity (M_w/M_n)	Am content (%)	X_{Am} (DP)	X_{Ap1} (DP)	X_{Ap2} (DP)	h_{Ap2}/h_{Ap1}	h_{Am}/h_{Ap1}
Pregel 1	2.30[a] ± 0.21	1.91[a] ± 0.20	28.7[a] ± 0.1	1738[b] ± 59	14.6[a] ± 0.0	37.6[b]± 0.1	0.54[a] ± 0.0	0.17[a] ± 0.01
Pregel 2	2.22[a] ± 0.23	2.06[a] ± 0.24	28.1[a] ± 0.8	1640[a] ± 44	14.2[a] ± 0.1	36.5[a] ± 0.2	0.55[a] ± 0.0	0.18[a] ± 0.01
Pregel 3	1.97[a] ± 0.17	1.91[a] ± 0.20	27.6[a] ± 0.7	1625[a] ± 66	14.4[a] ± 0.0	36.5[a] ± 0.6	0.58[a] ± 0.1	0.18[a] ± 0.01

Values with different letter between brackets for each column parameter denotes significant differences between starches at a significance level of $p < 0.05$.
M_w, weight-average molecular weight; M_n, number-average molecular weight; Polydispersity; ratio between M_w and M_n; Am and Ap stands for amylose and amylopectin, respectively. DP, Degree of polymerization; X_{Ap1}, DP of peak maximum of short (A + B1) amylopectin chains; X_{Ap2}, DP of peak maximum of long (B2 + B3) amylopectin chains; X_{Am}, DP of peak maximum of amylose chains; hAp2/hAp1, ratio between the height of Ap2 to Ap1 peak height in SEC weight molecular size distribution; ham/hap1, ratio between the height of Am to Ap1 peak height in SEC weight molecular size distribution.

Table S2. Peak maximum viscosity and final viscosity obtained from the pasting profiles of the pregelatinized drum-dried starches.

Sample	Cold Peak Viscosity (mPa·s)	Final Viscosity (mPa·s)
Pregel 1	8360[a] ± 43	1969[a] ± 13
Pregel 2	12833[b] ± 6	2185[b] ± 40
Pregel 3	14340[c] ± 50	3115[c] ± 33

Values with different letter between brackets for each column parameter denotes significant differences between starches at a significance level of $p < 0.05$.

Figure S1. Differential scanning calorimetry curves of drum-dried starches scanned at 10ºC/min from 25 to 95ºC after 24 h of hydration with a 1:3 starch:water ratio. Arrow indicates the presence of an endothermal transition in Pregel 1 starch presumably corresponding to the gelatinization of amylopectin double helices as previously reported for native wheat starch [45].

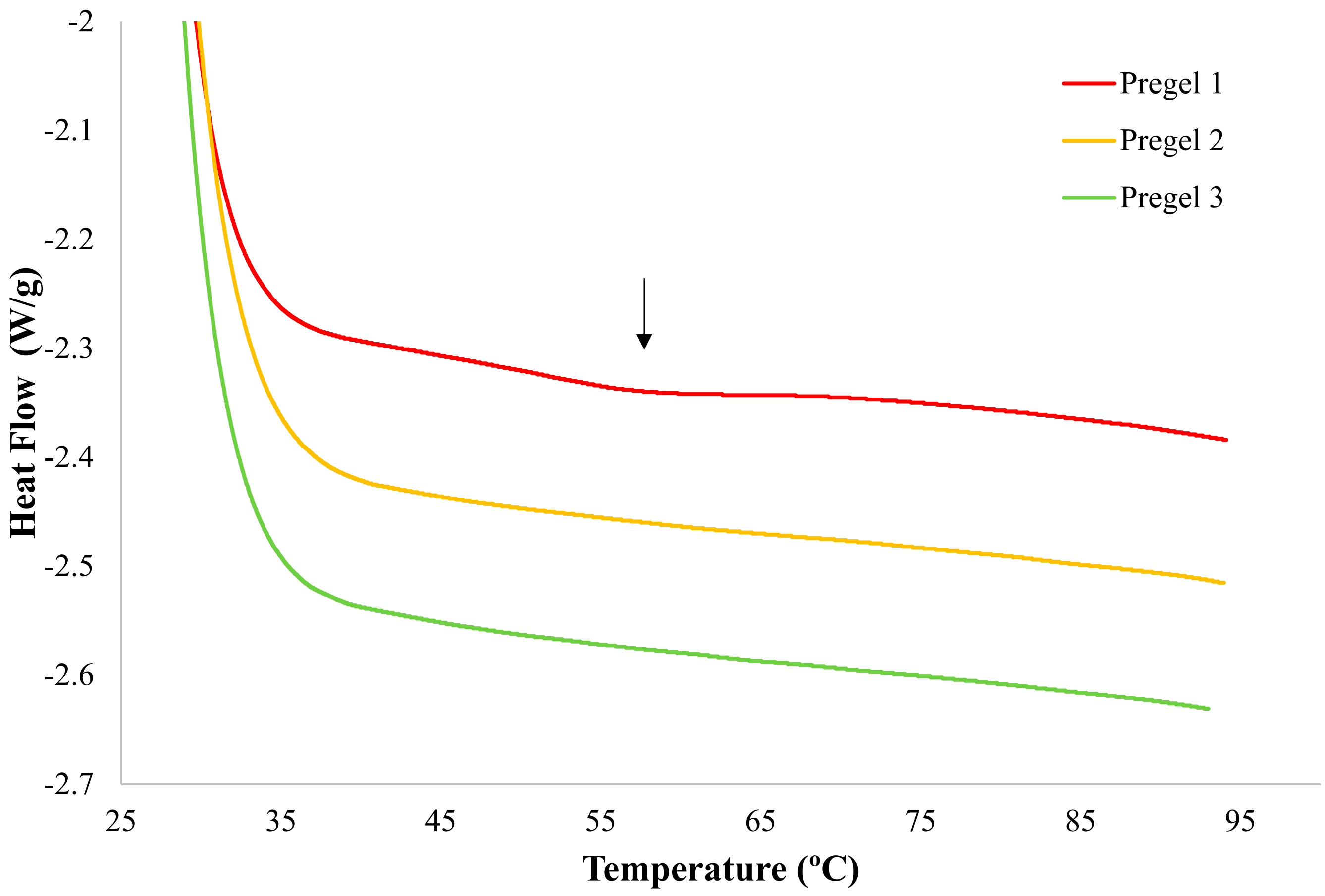

45. Sasaki, T., Matsuki, J. Effect of wheat starch structure on swelling power. *Cereal Chem.* **1998**, 75, 525–529.
